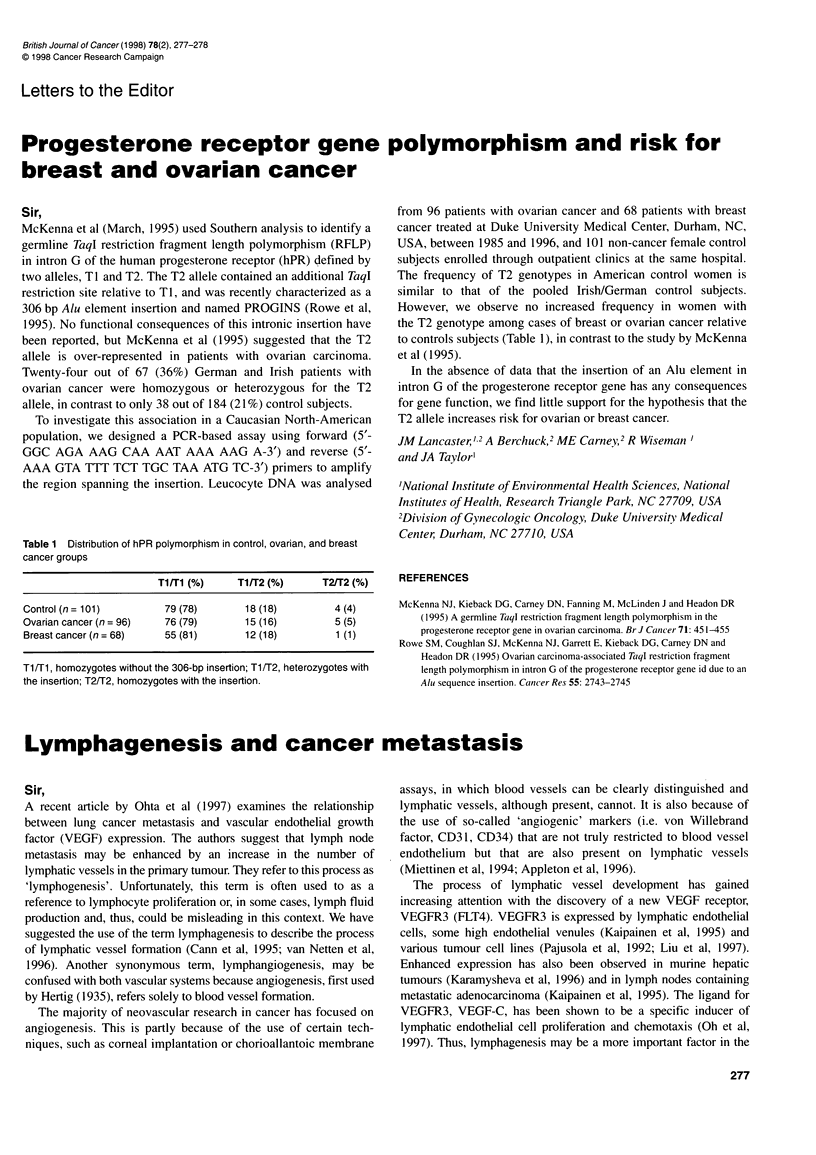# Progesterone receptor gene polymorphism and risk for breast and ovarian cancer.

**DOI:** 10.1038/bjc.1998.480

**Published:** 1998-07

**Authors:** J. M. Lancaster, A. Berchuck, M. E. Carney, R. Wiseman, J. A. Taylor


British Journal of Cancer (1998) 78(2), 277–278
© 1998 Cancer Research Campaign

Letters to the Editor

### Progesterone receptor gene polymorphism and risk for breast and ovarian cancer

**Sir,**

McKenna et al (March, 1995) used Southern analysis to identify a germline *Taq*I restriction fragment length polymorphism (RFLP) in intron G of the human progesterone receptor (hPR) defined by two alleles, T1 and T2. The T2 allele contained an additional *Taq*I restriction site relative to T1, and was recently characterized as a 306 bp *Alu* element insertion and named PROGINS (Rowe et al, 1995). No functional consequences of this intronic insertion have been reported, but McKenna et al (1995) suggested that the T2 allele is over-represented in patients with ovarian carcinoma. Twenty-four out of 67 (36%) German and Irish patients with ovarian cancer were homozygous or heterozygous for the T2 allele, in contrast to only 38 out of 184 (21%) control subjects.

To investigate this association in a Caucasian North-American population, we designed a PCR-based assay using forward (5′-GGC AGA AAG CAA AAT AAA AAG A-3′) and reverse (5′-AAA GTA TTT TCT TGC TAA ATG TC-3′) primers to amplify the region spanning the insertion. Leucocyte DNA was analysed from 96 patients with ovarian cancer and 68 patients with breast cancer treated at Duke University Medical Center, Durham, NC, USA, between 1985 and 1996, and 101 non-cancer female control subjects enrolled through outpatient clinics at the same hospital. The frequency of T2 genotypes in American control women is similar to that of the pooled Irish/German control subjects. However, we observe no increased frequency in women with the T2 genotype among cases of breast or ovarian cancer relative to controls subjects (Table 1), in contrast to the study by McKenna et al (1995).

In the absence of data that the insertion of an Alu element in intron G of the progesterone receptor gene has any consequences for gene function, we find little support for the hypothesis that the T2 allele increases risk for ovarian or breast cancer.

**Table 1** Distribution of hPR polymorphism in control, ovarian, and breast cancer groups

| | T1/T1 (%) | T1/T2 (%) | T2/T2 (%) |
|---|---|---|---|
| Control (*n* = 101) | 79 (78) | 18 (18) | 4 (4) |
| Ovarian cancer (*n* = 96) | 76 (79) | 15 (16) | 5 (5) |
| Breast cancer (*n* = 68) | 55 (81) | 12 (18) | 1 (1) |

T1/T1, homozygotes without the 306-bp insertion; T1/T2, heterozygotes with the insertion; T2/T2, homozygotes with the insertion.

*JM Lancaster,*[1,2] *A Berchuck,*[2] *ME Carney,*[2] *R Wiseman* [1] *and JA Taylor*[1]

[1]*National Institute of Environmental Health Sciences, National Institutes of Health, Research Triangle Park, NC 27709, USA*
[2]*Division of Gynecologic Oncology, Duke University Medical Center, Durham, NC 27710, USA*

**REFERENCES**

McKenna NJ, Kieback DG, Carney DN, Fanning M, McLinden J and Headon DR (1995) A germline *Taq*I restriction fragment length polymorphism in the progesterone receptor gene in ovarian carcinoma. *Br J Cancer* **71**: 451–455

Rowe SM, Coughlan SJ, McKenna NJ, Garrett E, Kieback DG, Carney DN and Headon DR (1995) Ovarian carcinoma-associated *Taq*I restriction fragment length polymorphism in intron G of the progesterone receptor gene id due to an *Alu* sequence insertion. *Cancer Res* **55**: 2743–2745